# Estimating thigh skeletal muscle volume using multi-frequency segmental-bioelectrical impedance analysis

**DOI:** 10.1186/s40101-021-00263-z

**Published:** 2021-09-30

**Authors:** Masashi Taniguchi, Yosuke Yamada, Masahide Yagi, Ryusuke Nakai, Hiroshige Tateuchi, Noriaki Ichihashi

**Affiliations:** 1 Human Health Sciences, Graduate School of Medicine, Kyoto University, 53, Kawahara-cho, Shogoin, Sakyo-ku, Kyoto, 606-8507 Japan; 2 National Institutes of Biomedical Innovation, Health and Nutrition, 1-23-1, Toyama, Shinjuku-ku, Tokyo, 162-8636 Japan; 3 Kokoro Research Center, Kyoto University, 53, Kawahara-cho, Shogoin, Sakyo-ku, Kyoto, 606-8507 Japan

**Keywords:** Segmental-bioimpedance analysis, Skeletal muscle volume, Thigh, Quadriceps, Estimation equation

## Abstract

**Background:**

The primary aim of this study was to investigate whether using the extracellular water/intracellular water (ECW/ICW) index and phase angle combined with segmental-bioimpedance analysis (BIA) improved the model fitting of skeletal muscle volume (SMV) estimation. The secondary aim was to compare the accuracy of segmental-BIA with that of ultrasound for estimating the quadriceps SMV measured with MRI.

**Methods:**

Seventeen young men (mean age, 23.8 ± 3.3 years) participated in the study. The T-1 weighted images of thigh muscles were obtained using a 1.5 T magnetic resonance imaging (MRI) scanner. Thigh and quadriceps SMVs were calculated as the sum of the products of anatomical cross-sectional area and slice thickness of 6 mm across all slices. Segmental-BIA was applied to the thigh region, and data on the 50-kHz bioelectrical impedance (BI) index, ICW index, ECW/ICW index, and phase angle were obtained. The muscle thickness index was calculated as the product of the mid-thigh muscle thickness, determined using ultrasound, and thigh length. The standard error of estimate (SEE) of the regression equation was calculated to determine the model fitting of SMV estimation and converted to %SEE by dividing the SEE values by the mean SMV.

**Results:**

Multiple regression analysis indicated that the combination of 50-kHz BI and the ECW/ICW index or phase angle was a significant predictor when estimating thigh SMV (SEE = 7.9 and 8.1%, respectively), but were lower than the simple linear regression (SEE = 9.4%). The ICW index alone improved the model fitting for the estimation equation (SEE = 7.6%). The model fitting of the quadriceps SMV with the 50-kHz BI or ICW index was similar to that with the skeletal muscle thickness index measured using ultrasound (SEE = 10.8, 9.6 and 9.7%, respectively).

**Conclusions:**

Combining the traditionally used 50-kHz BI index with the ECW/ICW index and phase angle can improve the model fitting of estimated SMV measured with MRI. We also showed that the model suitability of SMV estimation using segmental-BIA was equivalent to that on using ultrasound. These data indicate that segmental-BIA may be a useful and cost-effective alternative to the gold standard MRI for estimating SMV.

## Background

Skeletal muscle volume (SMV) is commonly used as a marker of muscle strength and physical function [1]. Age-associated loss in SMV has been identified as a key risk factor for falls, frailty, malnutrition and overall increased morbidity and mortality [2, 3]. Previous studies using whole-body and segmental measures of SMV have revealed associations between SMV and physical activity and/or exercise in young populations [4–6]. Furthermore, thigh muscles, especially quadriceps, are often the object of studies because of their importance in sports and daily living activities [7, 8]. Therefore, a non-invasive and cost-effective method for assessing the SMV of quadriceps may be useful in clinical settings.

Currently, imaging methods such as computed tomography (CT) and magnetic resonance imaging (MRI) are recognised as the gold standards for assessing SMV in clinical research. However, the high costs, time constraints, and limited access associated with these imaging approaches limit their applicability to the general population. In addition, the participants are subject to radiation exposure during CT measurements. One common alternative approach is dual-energy X-ray absorptiometry (DXA), which allows measurement of appendicular lean soft tissues (ALST), a reflection of SMV. Although DXA can be performed in a shorter amount of time and is more cost-effective than MRI and CT, limited access and radiation exposure limit the use of DXA.

In contrast, an alternative method for assessing SMV that is non-invasive, cost-effective, and suitable for both research and clinical purposes is bioelectrical impedance analysis (BIA). Several studies have reported on the validity of BIA in estimating SMV in young and old healthy populations when compared with CT, MRI, and DXA imaging [9–11]. To date, the single-frequency bioelectrical impedance (BI) index has been used to estimate SMV. However, recent developments incorporating theoretical models, where a multi-frequency approach for BIA combining a 50-kHz impedance index and the extracellular water (ECW) per intracellular water (ICW) index have enhanced the accuracy of ALST measurement [12, 13]. Additionally, the ECW/ICW index has been shown to be an indicator of muscle quality, as it represents the non-contractile tissue volume relative to the muscle cell volume [12]. Furthermore, the phase angle has also been associated with fat-free mass and muscle function [13, 14]. The phase angle concept is based on changes in resistance and reactance due to changes in the current as it passes through the tissues; thus, the measured phase angle reflects the resistant compartments of cellular membranes [15]. Therefore, the combination of the ECW/ICW index and the phase angle could further improve the estimation of SMV. However, no previous studies have investigated the combination of these parameters and their potential to enhance the accuracy of SMV assessment. To our knowledge, only one study has reported that the reactance of 50 kHz enhanced the accuracy of SMV in whole legs [11].

Moreover, the ICW index obtained using a multi-frequency segmental-BIA approach has been recognised as an indicator of muscle cell volume with high accuracy for estimating leg muscle mass [16] and is widely used [17–19]. The ICW index, defined as L^2^/Z_250-5_ (see the ‘Materials and methods’ section for equations), for both low and high frequencies, was considered when assessing muscle mass. A previous study [10] has reported that the ICW index using multi-frequency segmental-BIA is more accurate than the 50-kHz BI when estimating the ALST of thigh muscles. These data suggest that the ICW index may be a useful independent factor for estimating thigh SMV, when not accounting for electrical properties related to muscle quality, i.e. the ECW/ICW index and the phase angle.

Quadriceps SMV is an important clinical parameter when assessing performance levels in athletes and frailty and disability in older adults [7, 20, 21]. One limitation of BIA is that this approach does not allow measuring an individual’s muscles in a given area of measurements, and BIA assumes that the human body segment is a cylinder with a uniform tissue length and cross-sectional area. However, recent studies have shown that the electrical properties obtained from the quadriceps are associated with muscle strength during knee extension [17, 22]. These electrical properties of the thigh may, therefore, partially be explained by the quadriceps SMV. Yet, to date, no study has reported attempts to estimate the quadriceps SMV by segmental-BIA. Ultrasound is a commonly used approach in the clinic when estimating the SMV of the quadriceps based on muscle thickness [23, 24]. Segmental-BIA may offer an alternative approach, provided the accuracy is enhanced or comparable with ultrasound.

The primary aim of this study was to assess the effect of the ECW/ICW index or phase angle when measuring SMV. The secondary aim was to compare the model fitting of segmental-BIA with that of the ultrasound method for estimating the quadriceps SMV measured with MRI. We hypothesised that combining the 50-kHz BI index with the ECW/ICW index and phase angle would enhance the model fitting of thigh SMV measurements. Furthermore, we hypothesised that the ICW index would have a high accuracy independently of the ECW/ICW index and phase angle, and that segmental-BIA would be equivalent to ultrasound methods when measuring quadriceps SMV.

## Materials and methods

### Subjects

Seventeen young adult men (mean age, 23.8 ± 3.3 years; height, 170.9 ± 4.1 cm; body weight, 63.0 ± 3.7 kg) participated in the study. Eligible subjects were enrolled if they were healthy, not enrolled in an exercise programme, and could comply with all study procedures. Participants were excluded if they had a history of surgery or neuromuscular disorders in the lower extremities. All study procedures were approved by the Ethics Committee of the Kyoto University Graduate School of Medicine (R0881-3) and were conducted in accordance with the principles of the Declaration of Helsinki. All subjects provided written informed consent at enrolment.

On the basis of data from previous studies using imaging and electrical properties for the measurement of thigh muscles, we calculated the sample size required to achieve 80% power with an alpha (*α*) of 0.05 in our study [10]. To observe an effect size (*r*) of 0.73, a sample size of 12 subjects was estimated to be required for a linear regression analysis (G*Power 3.1.9.7, Universität Kiel, Germany).

### Experimental approach

The study protocol was based on a cross-sectional design. The subjects were instructed to refrain from vigorous exercise for 24 h before the assessments. On the day of the experiment, the subjects initially underwent an MRI scan of the thigh. Then, multi-frequency segmental-BIA and ultrasound measurements were conducted. All measurements were carried out after the subjects had rested in a supine position to take into account the immediate shift of body fluids.

### SMV measurement using MRI

The T-1 weighted images with a three-dimensional gradient-echo sequence of the thigh muscles on the right side of the leg were obtained using a 1.5 T MRI scanner (MAGNETOM Sonata; Siemens AG, Germany) with a body coil. This multi-slice sequence with a slice thickness of 3 mm was performed with acquisition parameters as follows: repetition time, 1940 ms; echo time, 2.6 ms; field of view, 400 × 250 mm; flip angle, 9°; voxel size, 1.6 × 1.6 × 3.0 mm. All MRI scans were conducted after subjects had rested in a supine position for > 15 min on the MRI bed. Transverse images going from the 12th thoracic vertebra to below the knee joint were obtained by connecting four measurement sections.

From each cross-sectional image, the region of interest in the quadriceps and hamstring muscles was traced using the OsiriX software (OsiriX MD; Pixmeo, Switzerland). Next, adipose and connective tissues within muscles were excluded with the help of the OsiriX 2D region growing method, which can highlight muscles and discard adipose and connective tissue based on pixel intensity thresholds. We adjusted the threshold values in each subject to exclude adipose and connective tissues as much as possible. The OsiriX software can automatically convert the number of voxels and voxel size within the region of interest to the anatomical cross-sectional area (cm^2^) except for adipose and connective tissues. This procedure was performed once in every two images throughout the whole muscle length, going from the most proximal to the most distal images. Then, the volume (cm^3^) in each slice was calculated by multiplying the anatomical cross-sectional area by the slice thickness (6 mm). Finally, the SMV of each muscle was obtained by summing the volume of all the slices. The SMV of the thigh was defined here as the sum of the quadriceps and hamstrings. All imaging analysis was performed by the same well-trained investigator.

### Measurement of thigh muscle electrical properties using multi-frequency segmental-BIA

**Fig. 1:**
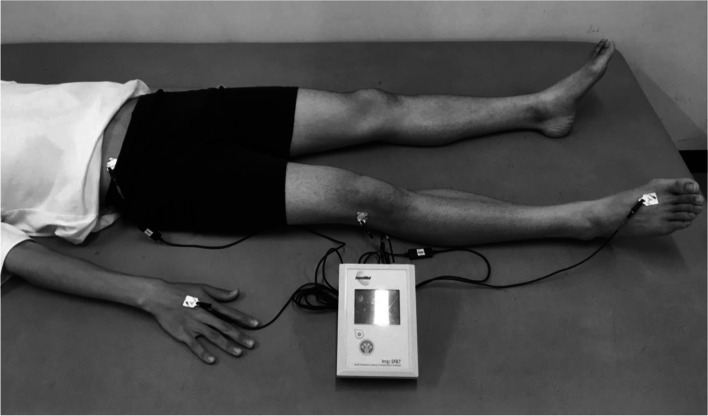
The measurement setting of segmental-bioelectrical impedance analysis in the thigh

Multi-frequency segmental-BIA was conducted after the subjects lying in a relaxed supine position for > 5 min to account for the immediate shift of body fluids [25]. Segmental-BIA of the right thigh was performed for a logarithmic spectrum of 256 frequencies ranging from 4 to 1000 kHz (SFB7; ImpediMed Inc., Australia) using disposable clip-type electrodes (Red Dot TM; 3M Inc., Japan). After the electrode sites were cleaned with alcohol, disposable electrodes were placed on the four sites as previously described [19]. Two current-carrying electrodes were placed on the dorsal surfaces of the hand and foot on the right side, and two sensing electrodes were placed on the anterior superior iliac spine and lateral knee articular condyles (Fig. 1). Three consecutive repetitions for measurements of segmental-BIA were conducted to obtain the BI. The distance between the anterior superior iliac spine and the proximal end of the patella was measured as the segmental length (L; cm).

Data processing was performed using the SFB7 Bioimp software (ImpediMed Inc., Australia). The resistance at 5, 50 and 250 kHz was obtained by extrapolation after fitting the spectrum of bioelectrical data with the Cole model. The 50-kHz BI index of the thigh segment was calculated as L^2^/Z_50_ (cm^2^/Ω). The impedance measurements at low-frequency current (≤ 50 kHz) predominantly reflected the ECW, and the impedance measurements at high-frequency current (≥ 250 kHz) predominantly reflected the ICW. The impedance for the ECW was calculated as L^2^/Z_5_ (cm^2^/Ω). The impedance of the ICW compartment (Z_250_-_5_) was calculated as 1/[(1/Z_250_) − (1/Z_5_)], and the impedance for the ICW (i.e. ICW index) was calculated as L^2^/Z_250_-_5_ (cm^2^/Ω). The ECW/ICW index of the thigh segment was calculated as Z_5_/Z_250_-_5_. The phase angle at 50 kHz was also obtained from the Cole model. The mean values of three repetitions were subsequently used in analysis. The between-day coefficients of variation and standard error mean were 3.0% and 0.047, respectively.

### Measurement of thigh muscle thickness using ultrasound

All measurements using ultrasound were conducted with subjects in the supine position. The transverse ultrasound images were obtained using a B-mode ultrasonography device with liner probe (Aixplorer, Super Sonic Imagine, France). Ultrasound settings were the same for all measurements and were established by the same investigator. The muscle thickness of the thigh was defined as the sum of the rectus femoris and vastus intermedius at the midpoint between the anterior superior iliac spine and the proximal end of the patella. The between-day coefficients of variation and standard error mean for repeated muscle thickness measurements was 1.2% and 0.106, respectively. Previous research has indicated that muscle length influences SMV estimation [23]. To account for this, the distance between the greater trochanter of femur and lateral knee articular condyles was measured as the thigh length. The thigh length was obtained using a tape measure in the relaxed supine position and rounded to the nearest 0.5 cm. Subsequently, the muscle thickness index was calculated as the product of muscle thickness and thigh length.

### Statistical analysis

All data are presented as mean (± standard deviation (SD)), and all data were parametric. Correlations between the SMVs, 50-kHz BI index, ICW index, ECW/ICW index, phase angle, muscle thickness index and physical characteristics (age, height and body weight) were analysed using Pearson’s correlation coefficients. Simple linear regression analyses were performed to determine (1) the relationship between the thigh SMV and 50-kHz BI index or ICW index and (2) the relationship between the quadriceps SMV and 50 kHz BI index, ICW index or muscle thickness index. The standard error of estimate (SEE) of the regression equation was calculated to determine the model fitting of SMV estimation for each variable. SEE was then converted to %SEE by dividing the SEE values by the mean SMV. To examine the effects of the ECW/ICW index or phase angle for estimating SMV, we also conducted multiple linear regression analyses with the 50-kHz BI index and ECW/ICW index (model 1) or phase angle (model 2), the ICW index and ECW/ICW index (model 3), or the phase angle (model 4) as an independent variable and thigh SMV as the dependent variable. Previous data [14] have shown that the phase angle could be predicted by the ECW/ICW index (beta, *β* = −0.847). Thus, we applied these data individually in the regression models. Similarly, multiple linear regression analyses were applied to the quadriceps SMV as the dependent variable. We confirmed the presence of a multicollinearity effect by calculating the variance inflation factor values. All statistical analyses were carried out using the SPSS software (version 25.0; SPSS Japan Inc., Japan). The level of significance was set at *p* < 0.05.

## Results

**Fig. 2:**
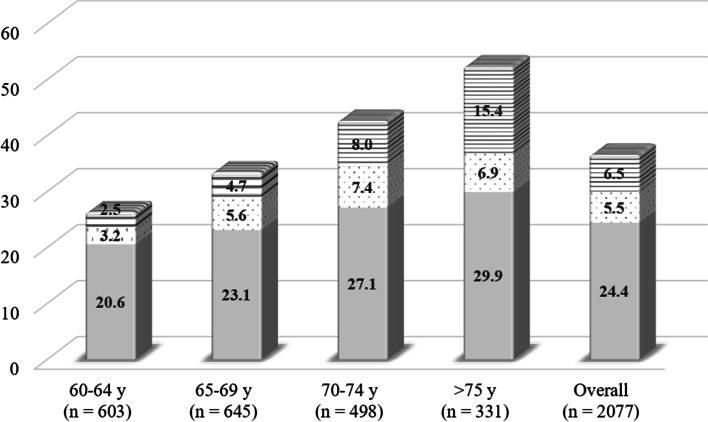
Simple linear regression for estimating thigh skeletal muscle volume (SMV) measured by magnetic resonance imaging (MRI) with 50-kHz bioelectrical impedance index (BI) (**A**) and intracellular water (ICW) index (**B**). (**A**) *y* = 54.8 × 50 kHz BI index + 578.3; *R*^2^ = 0.548; SEE = 239.0 cm^3^, 9.4%. (**B**) *y* = 119.5 × ICW index + 927.5; *R*^2^ = 0.703; SEE = 193.7 cm^3^, 7.6%

**Fig. 3:**
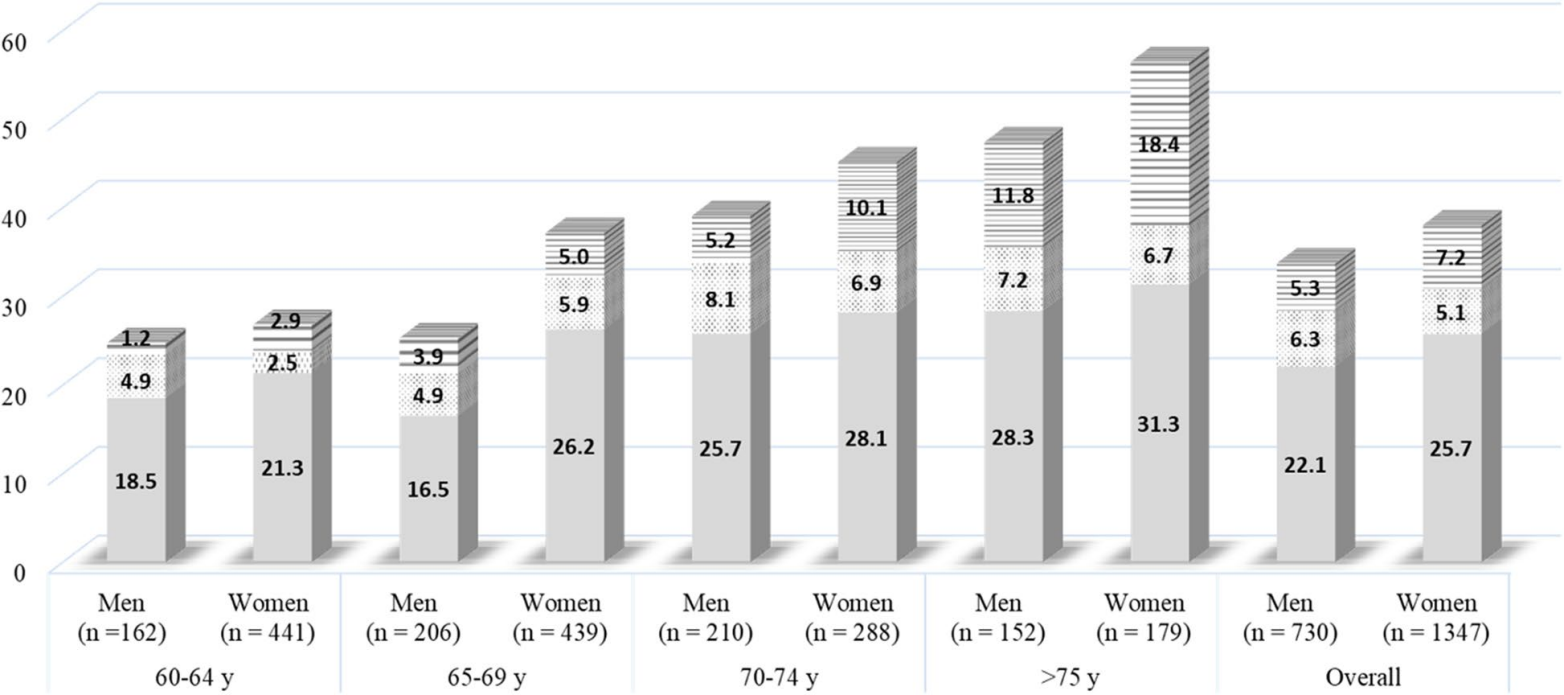
Simple linear regression for estimating quadriceps skeletal muscle volume (SMV) measured using magnetic resonance imaging (MRI). Simple linear regression relationship between quadriceps SMV using 50-kHz bioimpedance index (BI) (**A**), intracellular water index (ICW) (**B**), and muscle thickness index (**C**). (**A**) *y* = 36.1 × 50 kHz BI index + 545.7; *R*^2^ = 0.433; SEE = 198.5 cm^3^, 10.8%. (**B**) *y* = 78.4 × ICW index + 779.5; *R*^2^ = 0.551; SEE = 176.6 cm^3^, 9.6%. (**C**) *y* = 10.3 × muscle thickness index + 35.3; *R*^2^ = 0.543; SEE = 178.1 cm^3^, 9.7%

The SMV, electrical properties and muscle thickness index of the subjects are presented in Table 1. Table 2 presents the correlation coefficients between the SMV, electrical properties, muscle thickness index and physical characteristics of the participants. There were positive correlations between the thigh and quadriceps SMV and 50-kHz BI index, ICW index, phase angle, muscle thickness index and body weight and a significant negative correlation with the ECW/ICW index. The 50-kHz BI index was not significantly correlated with the ECW/ICW index or phase angle. In contrast, there was a significant negative correlation between the ICW index and ECW/ICW index and a significant positive correlation between ICW index and phase angle. Moreover, there was a significant positive correlation between the ECW/ICW index and phase angle (Table 2). The SEE between thigh SMV and the 50-kHz BI index or ICW index were 239.0 cm^3^ (9.4%) and 193.7 cm^3^ (7.6%), respectively (Fig. 2). In addition, the SEE between the quadriceps SMV and the 50-kHz BI index, ICW index or muscle thickness index was 198.5 cm^3^ (10.8%), 176.6 cm^3^ (9.6%) and 178.1 cm^3^ (9.7%), respectively (Fig. 3).

**Table 1:** Characteristics of skeletal muscle volume (SMV), electrical properties, and muscle thickness index

Variable	Mean (± SD)	Minimum	Maximum
**Thigh SMV (cm**^**3**^**)**	2540 (± 344)	1968	3076
**Quadriceps SMV (cm**^**3**^**)**	1838 (± 255)	1376	2215
**50 kHz BI index (cm**^**2**^**/Ω)**	35.8 (± 4.7)	27.6	44.8
**ICW index (cm**^**2**^**/Ω)**	13.5 (± 2.4)	9.0	18.7
**ECW/ICW index (a.u)**	2.2 (± 0.24)	1.7	2.8
**Phase angle (degree)**	9.3 (± 0.8)	7.6	11.1
**Muscle thickness index (cm**^**2**^**)**	176 (± 18)	127	200

*SD* standard deviation, *SMV* skeletal muscle volume, *BI* bioelectrical impedance, *ICW* intracellular water, *ECW/ICW* extracellular water per intracellular water

**Table 2:** Correlation coefficients between skeletal muscle volume (SMV), electrical properties, muscle thickness index, and physical characteristics

Variable	Thigh SMV (cm^**3**^)	Quadriceps SMV (cm^**3**^)	50-kHz BI index (cm^**2**^/Ω)	ICW index (cm^**2**^/Ω)	ECW/ICW index (a.u)	Phase angle (degree)	Muscle thickness index (cm^**3**^)	Age (years)	Height (cm)	Body weight (kg)
**Thigh SMV (cm**^**3**^**)**	-	0.951**	0.740**	0.839*	−0.600*	0.571*	0.771**	−0.178	0.121	0.540*
**Quadriceps SMV (cm**^**3**^**)**		-	0.658**	0.743**	−0.532**	0.484*	0.737**	−0.109	0.117	0.555*
**50-kHz BI index (cm**^**2**^**/Ω)**			-	0.878*	−0.308	0.288	0.556**	−0.234	0.275	0.323
**ICW index (cm**^**2**^**/Ω)**				-	−0.712**	0.695**	0.627**	−0.298	0.095	0.332
**ECW/ICW index (a.u)**					-	−0.959**	−0.455	0.251	0.217	−0.281
**Phase angle (degree)**						-	0.506*	−0.224	−0.211	0.220
**Muscle thickness index (cm**^**3**^**)**							-	−0.109	0.332	0.646*
**Age (years)**								-	0.135	0.278
**Height (cm)**									-	0.392
**Body weight (kg)**										

*SMV* skeletal muscle volume, *BI* bioelectrical impedance, *ICW* intracellular water, *ECW/ICW* extracellular water per intracellular water

*p < 0.05

**p < 0.01

The multiple linear regression analysis showed that the 50-kHz BI index and ECW/ICW index were significant independent predictors for estimating the thigh SMV (model 1, Table 3). The phase angle was also identified as a significant predictor for thigh SMV (model 2, Table 3). The SEE were 201.2 cm^3^ (7.9%) in model 1 and 205.4 cm^3^ (8.1%) in model 2, which were both lower than the SEE obtained in the simple linear regression (Table 3 and Fig. 2). In contrast, the ICW index was only a significant independent predictor for estimating the thigh SMV (models 3 and 4, Table 3), and the SEE in these models was not higher than those in simple linear regression. The variance inflation factors ranged from 1.091 to 2.031 in models 1 to 4, suggesting no multicollinearity effects in the models.

**Table 3:** Coefficients of multiple linear regression analysis for estimating thigh and quadriceps skeletal muscle volume (SMV)

	Thigh SMV (cm^**3**^)				Quadriceps SMV (cm^**3**^)		
	Non-standardised (***B***)	Standardised (***β***)	***p value***		Non-standardised (***B***)	Standardised (***β***)	***p*** value
**Model 1**	*R*^2^ = 0.701, SEE = 201.2 cm^3^ (7.9%)			**Model 1**	*R*^2^ = 0.552, SEE = 182.5 cm^3^ (9.9%)		
Constant	2180.6		0.009	Constant	1595.5		0.027
50-kHz BI index (cm^2^/Ω)	45.4	0.614	0.001	50-kHz BI index (cm^2^/Ω)	29.9	0.546	0.012
ECW/ICW index (a.u)	−581.2	−0.411	0.018	ECW/ICW index (a.u)	−380.8	−0.363	0.074
**Model 2**	*R*^2^ = 0.689, SEE = 205.4 cm^3^ (8.1%)			**Model 2**	*R*^2^ = 0.528, SEE = 188.5 cm^3^ (10.3%)		
Constant	-673.7		0.309	Constant	−217.9		0.714
50-kHz BI index (cm^2^/Ω)	46.4	0.627	0.001	50-kHz BI index (cm^2^/Ω)	31.0	0.565	0.011
Phase angle (degree)	167.5	0.391	0.025	Phase angle (degree)	102.2	0.322	0.115
**Model 3**	*R*^2^ = 0.703, SEE = 200.5 cm^3^ (7.9%)			**Model 3**	*R*^2^ = 0.551, SEE = 182.8 cm^3^ (9.9%)		
Constant	951.7		0.341	Constant	796.6		0.381
ICW index (cm^2^/Ω)	119.0	0.835	0.001	ICW index (cm^2^/Ω)	78.0	0.739	0.012
ECW/ICW index (a.u)	−7.7	-0.005	0.979	ECW/ICW index (a.u)	−5.4	−0.005	0.984
**Model 4**	*R*^2^ = 0.704, SEE = 200.4 cm^3^ (7.9%)			**Model 4**	*R*^2^ = 0.553, SEE = 182.4 cm^3^ (9.9%)		
Constant	989.8		0.125	Constant	900.2		0.125
ICW index (cm^2^/Ω)	121.8	0.855	0.001	ICW index (cm^2^/Ω)	82.9	0.785	0.007
Phase angle (degree)	−10.1	−0.024	0.909	Phase angle (degree)	−19.6	−0.061	0.808

*SMV* skeletal muscle volume, *BI* bioelectrical impedance, *ICW* intracellular water, *ECW/ICW* extracellular water per intracellular water, *SEE* standard error of the estimate

The 50-kHz BI index and ICW index were significant independent predictors for estimating the quadriceps SMV, but not the ECW/ICW index and phase angle (Table 3). The SEE values in the models used for estimating the quadriceps SMV using electrical properties ranged from 182.4 to 188.5 cm^3^ (9.9% to 10.3%; Table 3). These SEE values were similar to those in the simple linear regression between the quadriceps SMV and the muscle thickness index (178.1 cm^3^, 7.6%; Fig. 3).

Based on the SEE values, we defined the estimation equations for the thigh and quadriceps SMV as follows:

1 $$\mathrm{Thigh}\ \mathrm{SMV}\ \left({\mathrm{cm}}^3\right)=45.4\times 50-\mathrm{kHz}\ \mathrm{BI}\ \mathrm{index}-581.2\times \mathrm{ECW}/\mathrm{ICW}\ \mathrm{index}+2180.6$$

or

2 $$\mathrm{Thigh}\ \mathrm{SMV}\ \left({\mathrm{cm}}^3\right)=46.4\times 50-\mathrm{kHz}\ \mathrm{BI}\ \mathrm{index}+167.5\times \mathrm{phase}\ \mathrm{angle}-673.7$$

or

3 $$\mathrm{Thigh}\ \mathrm{SMV}\ \left({\mathrm{cm}}^3\right)=119.5\times \mathrm{ICW}\ \mathrm{index}+927.5$$

and

4 $$\mathrm{Quadriceps}\ \mathrm{SMV}\ \left({\mathrm{cm}}^3\right)=78.4\times \mathrm{ICW}\ \mathrm{index}+779.5$$

or

5 $$\mathrm{Quadriceps}\ \mathrm{SMV}\ \left({\mathrm{cm}}^3\right)=10.3\times \mathrm{muscle}\ \mathrm{thickness}\ \mathrm{index}+35.3$$

## Discussion

To the best of our knowledge, this is the first study to verify the contributions of the ECW/ICW index or phase angle for estimating the thigh SMV when measured by MRI. We found that the ICW index alone enhanced the model fitting of SMV estimation when compared with the ECW/ICW index and the phase angle approach, confirming our original hypothesis. These data suggested that the combination of the 50-kHz BI index and ECW/ICW index or the ICW index alone can accurately estimate the thigh SMV. We also found, in the sample studied here, that the 50-kHz BI index and ICW index could predict the quadriceps SMV but not the ECW/ICW index and phase angle. However, we demonstrated that the SEEs from the estimation equations for segmental-BIA and ultrasound were equivalent in the model fitting, suggesting that segmental-BIA may provide an alternative method to ultrasound when estimating the quadriceps SMV measured with MRI.

Traditionally, body composition has been measured using single-frequency BIA (predominantly at 50 kHz). An earlier study [9] reported that the 50-kHz BI index was strongly correlated with the thigh SMV measured by MRI. Since the measurement site on the thigh used for obtaining the 50-kHz BI in our study varied from that in the previous study, we had to assess the %SEE values, which indicated model fitting, and not the estimation equations. As in the previous study, in which the SEE value was 362.3 cm^3^ (10.4%), we found a significant correlation between the thigh SMV and the 50-kHz BI index, with a SEE value of 239.0 cm^3^ (9.4%). However, these results must be interpreted with caution as the lower frequency current of 50 kHz may not pass through cells sufficiently, leading to overestimation of the thigh SMV in subjects with a high ECW/ICW ratio [10]. In the present study, the multiple regression analysis indicated that the combination of the 50-kHz BI and ECW/ICW index was independently associated with the thigh SMV, and that the model fitting was improved (SEE = 201.2 cm^3^, 7.9%) when compared with that on using the 50-kHz BI index alone. These data suggested that the addition of the ECW/ICW index with lower frequencies may improve accuracy when estimating the SMV. It should be noted that a relaxed position is required before and during the BIA measurement since the ECW/ICW index fluctuates with muscle contraction [19]. Today, many BIA devices can measure the impedance value at frequencies of 5, 50, and 250 kHz. The use of the ECW/ICW index, which simply calculates the impedance value at 5 and 250 kHz, is a reasonable and innovative method to help improve estimation accuracy.

Muscle quality is commonly assessed using the ECW/ICW index, as it has been shown to account for the non-contractile parts of the muscle [26]. A previous study has reported that the ECW/ICW index and the 50-kHz BI calculated using electrical properties represent muscle quality and muscle quantity, respectively, suggesting that these are independent factors that may act as biomarkers to reflect changes in muscle composition [22]. In fact, our results showed that the 50-kHz BI index was not significantly correlated with the ECW/ICW index. In this study, SMV was calculated by excluding adipose and connective tissues within muscles based on pixel intensity on the MRI images. This allowed us to determine that the ECW/ICW index of standardised coefficients for estimating the thigh SMV was negative (*β* = −0.411), which was confirmed using a theoretical interpretation of the estimation equation. Non-contractile tissue in lower limb muscles has been reported to range between 5 to 7% and 7 to 19% in young and older adults [27–29]. Furthermore, an increase in the ECW/ICW index has been shown to correlate with age [10, 30]. These findings suggested that the ECW/ICW index assessed by BIA may be useful when estimating age-related changes in SMV in older populations. However, further research is warranted to identify at what point in life age-associated changes occur.

A previous study [31] found that the phase angle was correlated with age. From an electrical standpoint, the theory surrounding the phase angle is based on the changes in resistance and reactance that occur as alternating currents penetrate cells. The observed phase shifts have been shown to reflect the size of the cells, the permeability of the cell membrane and the fluid distribution of the tissues [32] and therefore altogether reflect the volume of various tissue compartments and the hydration status [15]. Furthermore, in a recent study [14], the total-body water content was measured through the ICW by heavy hydrogen isotope dilution and total-body potassium counting methods to calculate the ECW/ICW index of the whole body. The authors indicated that the wrist-to-ankle phase angle was explained by sex, age, height, BMI, fat-free mass, ethnicity and the ECW/ICW index. They also reported that the ECW/ICW index was associated with the phase angle, consistent with our correlation analysis results. This was also the rationale for incorporating the phase angle and ECW/ICW index into the multiple linear regression analyses models. Altogether, the data from this study indicated that the model fitting of the thigh SMV estimation could be improved when combining the ECW/ICW index or the phase angle with the traditional single-frequency BIA. However, our data also revealed that the ICW index alone gave a better fit than that on incorporating the ECW/ICW index or the phase angle when estimating the thigh SMV, as shown by the SEE value (193.7 cm^3^, 7.6%). The better model fit of the ICW index alone was likely to be because of the use of low- and high-frequency impedance currents during the multi-frequency segmental-BIA, providing an indication of muscle cell mass, reflecting SMV. Our findings indicated that the ICW index can effectively improve the accuracy of estimating SMV and may prove to be a useful tool in clinical settings.

Using simple linear regressions, we found a moderate-to-strong association between the quadriceps SMV and the 50-kHz BI and the ICW index (SEE values, 198.5 cm^3^ (10.8%) and 176.6 cm^3^ (9.6%), respectively). Generally, the use of BIA when estimating the SMV was based on the assumption that the human body segment is a cylindrical conductor. However, this does not take into account the fact that the quadriceps make up just over a half of the whole thigh volume (54.5 to 56%), which may increase the estimation error when estimating the SMV of the quadriceps [33]. Furthermore, the addition of the ECW/ICW and phase angle did not enhance the model fitting when estimating the quadriceps SMV. Age-associated changes in the phase angle have generally been observed starting in middle age [31, 34]. The small degree of degeneration in the quadriceps of younger subjects may not be associated with this prediction model. Nevertheless, the fit of the estimation model for the quadriceps SMV using the ICW index as part of the segmental-BIA was equivalent to that of the ultrasound approach (SEE = 178.1 cm^3^, 9.7%), although the 50-kHz BI had a slightly low value. The fit of the estimation model using segmental-BIA in this study was not inferior to that of the ultrasound approach, even when compared with previous studies that estimated the quadriceps SMV using ultrasound (SEE = 198.5cm^3^, 11.1%) [35]. Therefore, these findings suggest segmental-BIA as an alternative method to ultrasound when assessing the quadriceps SMV.

This study has several limitations. First, the use of our male cohort with an age range of 23‑27 years to generate the estimation equations for SMV makes any generalisation difficult. Previous studies have shown that age, sex and body weight are likely to affect estimation equations for the SMV [12, 36, 37]. Although the SMVs of thigh and quadriceps muscles significantly correlated with body weight, we did not include body weight in the estimation equations. Multiple-BIA has the advantages of distinguishing muscle mass and non-contractile tissue mass from body weight; therefore, we tried to determine the SMV estimation equations that were not affected by body weight. Further work is warranted in a larger cohort with a broader range of ages (old, middle and young) and body weights and including both male and female participants to develop estimation equations that may enhance the model fitting when estimating SMV and can be generalised to a broad range of populations. Second, although segmental-BIA has been applied to a small region within the same limb in a recent study [38], this approach cannot distinguish individual muscles. Thus, it could be considered that the estimation equation of quadriceps SMV using the impedance index includes the effect of other thigh muscles. Finally, although the study was suitably powered to develop the estimation equations, no cross-validation was conducted. The estimation equations proposed here warrant further validation to establish whether they can be appropriately generalised to similar and broader populations.

## Conclusions

We demonstrated that the traditionally used 50-kHz BI index combined with the ECW/ICW index and phase angle can improve the estimation model of the SMV measured with the gold standard MRI. We also showed that the ICW index alone was an effective predictor of the thigh and quadriceps SMVs. Finally, we demonstrated that the model suitability of the SMV estimation determined using multi-frequency segmental-BIA was equivalent to that obtained using ultrasound methods. Taken together, these data indicated that segmental-BIA may be a useful and cost-effective alternative to the gold standard MRI for estimating the SMV.

## Data Availability

The datasets used and/or analysed during the study are available from the corresponding author on reasonable request.

## Abbreviations

ALST: Appendicular lean soft tissues
BI: Bioelectrical impedance
BIA: Bioelectrical impedance analysis
CT: Computerised tomography
DXA: Dual-energy X-ray absorptiometry
ECW: Extracellular water
ICW: Intracellular water
L: Length
MRI: Magnetic resonance imaging
SD: Standard deviation
SEE: Standard error of the estimate
SMV: Skeletal muscle volume
