# Correction to: Estimating thigh skeletal muscle volume using multi-frequency segmental-bioelectrical impedance analysis

**DOI:** 10.1186/s40101-021-00270-0

**Published:** 2021-12-13

**Authors:** Masashi Taniguchi, Yosuke Yamada, Masahide Yagi, Ryusuke Nakai, Hiroshige Tateuchi, Noriaki Ichihashi

**Affiliations:** 1 Human Health Sciences, Graduate School of Medicine, Kyoto University, 53, Kawahara-cho, Shogoin, Sakyo-ku, Kyoto, 606-8507 Japan; 2 National Institutes of Biomedical Innovation, Health and Nutrition, 1-23-1, Toyama, Shinjuku-ku, Tokyo, 162-8636 Japan; 3 Kokoro Research Center, Kyoto University, 53, Kawahara-cho, Shogoin, Sakyo-ku, Kyoto, 606-8507 Japan

**Correction to: J Physiol Anthropol 40, 13 (2021)**

**https://doi.org/10.1186/s40101-021-00263-z**

Following the publication of the original article [1] the authors noticed that the published Figs. 2 and 3 are incorrect. These were not replaced during corrections stage.

The original article [1] has been updated.

Below are the correct Figs. 2 and 3.

**Fig. 2:**
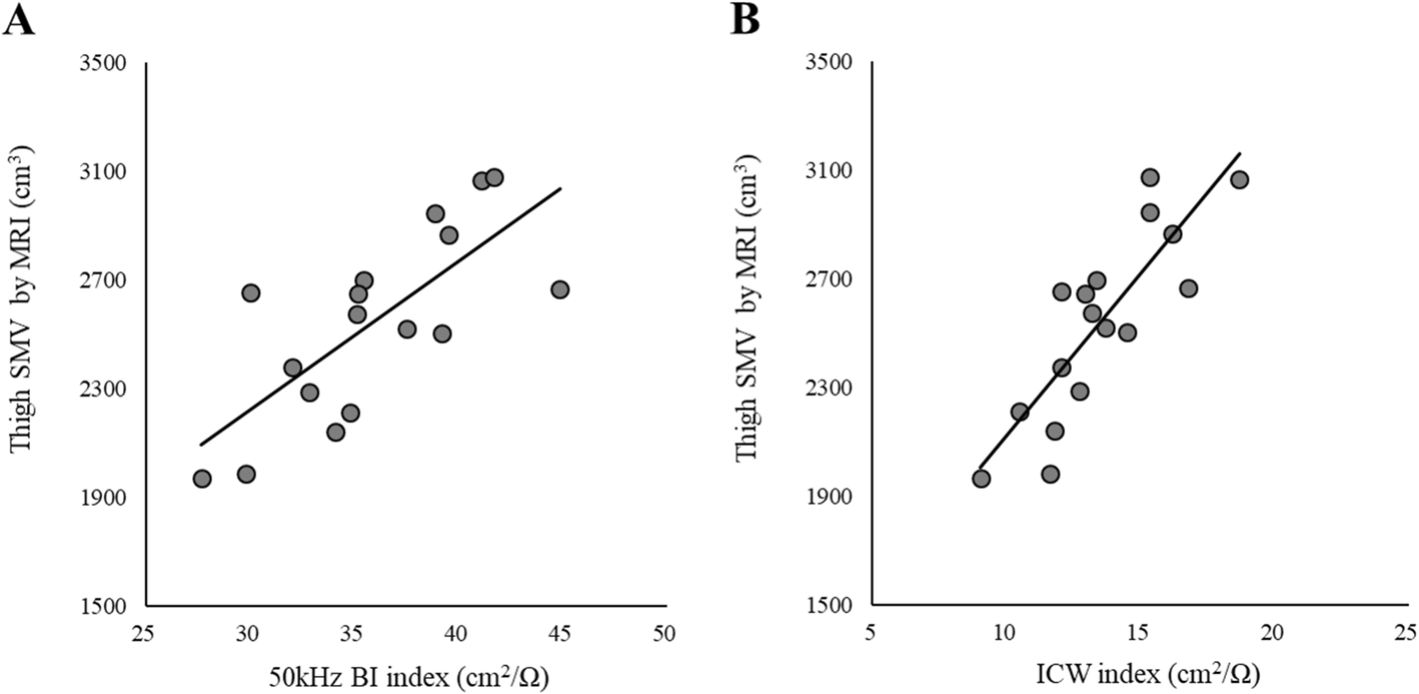
Simple linear regression for estimating thigh skeletal muscle volume (SMV) measured by magnetic resonance imaging (MRI) with 50-kHz bioelectrical impedance index (BI) (**A**) and intracellular water (ICW) index (**B**). **A** *y* = 54.8 × 50 kHz BI index + 578.3; *R*^2^ = 0.548; SEE = 239.0 cm^3^, 9.4%. **B** *y* = 119.5 × ICW index + 927.5; *R*^2^ = 0.703; SEE = 193.7 cm^3^, 7.6%

**Fig. 3:**
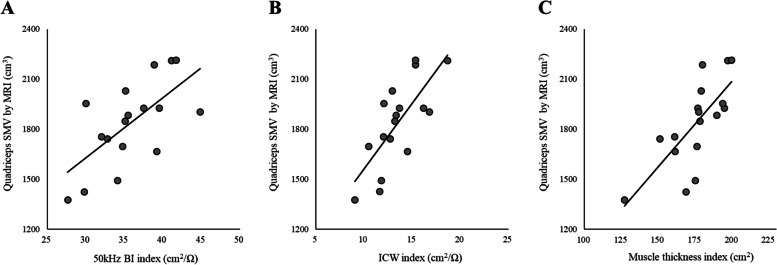
Simple linear regression for estimating quadriceps skeletal muscle volume (SMV) measured using magnetic resonance imaging (MRI). Simple linear regression relationship between quadriceps SMV using 50-kHz bioimpedance index (BI) (**A**), intracellular water index (ICW) (**B**), and muscle thickness index (**C**). **A** *y* = 36.1 × 50 kHz BI index + 545.7; *R*^2^ = 0.433; SEE = 198.5 cm^3^, 10.8%. **B** *y* = 78.4 × ICW index + 779.5; *R*^2^ = 0.551; SEE = 176.6 cm^3^, 9.6%. **C** *y* = 10.3 × muscle thickness index + 35.3; *R*^2^ = 0.543; SEE = 178.1 cm^3^, 9.7%
